# Robotic colorectal surgery for laparoscopic surgeons with limited experience: preliminary experiences for 40 consecutive cases at a single medical center

**DOI:** 10.1186/s12893-015-0057-6

**Published:** 2015-06-18

**Authors:** Ching-Wen Huang, Yung-Sung Yeh, Cheng-Jen Ma, Tak-Kee Choy, Ming-Yii Huang, Chun-Ming Huang, Hsiang-Lin Tsai, Wen-Hung Hsu, Jaw-Yuan Wang

**Affiliations:** Division of Gastroenterology and General Surgery, Department of Surgery, Kaohsiung Medical University Hospital, Kaohsiung Medical University, Kaohsiung, 807 Taiwan; Department of Surgery, Kaohsiung Municipal Hsiao-Kang Hospital, Kaohsiung Medical University, Kaohsiung, Taiwan; Graduate Institute of Medicine, College of Medicine, Kaohsiung Medical University, Kaohsiung, Taiwan; Division of Trauma, Department of Surgery, Kaohsiung Medical University Hospital, Kaohsiung Medical University, Kaohsiung, Taiwan; Graduate Institute of Clinical Medicine, College of Medicine, Kaohsiung Medical University, Kaohsiung, Taiwan; Division of Colorectal Surgery, Department of Surgery, Yuan’s General Hospital, Kaohsiung, Taiwan; Department of Radiation Oncology, Kaohsiung Medical University Hospital, Kaohsiung Medical University, Kaohsiung, Taiwan; Cancer Center, Kaohsiung Medical University Hospital, Kaohsiung Medical University, Kaohsiung, Taiwan; Department of Radiation Oncology, Faculty of Medicine, College of Medicine, Kaohsiung Medical University, Kaohsiung, Taiwan; Division of General Surgery Medicine, Department of Surgery, Kaohsiung Medical University Hospital, Kaohsiung Medical University, Kaohsiung, Taiwan; Division of Gastroenterology, Department of Internal Medicine, Kaohsiung Medical University Hospital, Kaohsiung Medical University, Kaohsiung, Taiwan; Department of Internal Medicine, Faculty of Medicine, College of Medicine, Kaohsiung Medical University, Kaohsiung, Taiwan; Department of Surgery, Faculty of Medicine, College of Medicine, Kaohsiung Medical University, Kaohsiung, Taiwan; Center for Biomarkers and Biotech Drugs, Kaohsiung Medical University, Kaohsiung, Taiwan

**Keywords:** Robotic colorectal surgery, Da Vinci® Surgical System, Colorectal cancer, Intersphincteric resection, Lower anterior resection

## Abstract

**Background:**

We present our preliminary experiences and results for forty consecutive patients with colorectal cancer (CRC) who were treated by robotic surgery.

**Methods:**

Between May 2013 and September 2014, forty patients with CRC received robotic surgery at a single institution. The clinicopathological features and perioperative parameters were retrospectively analyzed.

**Results:**

Of the 40 patients with CRC, 33 (82.5 %) had rectal cancers, and 22 (66.7 %) of those 33 patients also underwent pre-operative concurrent chemoradiotherapy (CCRT). The two most frequent surgical procedures were intersphincteric resection (ISR) with coloanal anastomosis (16/40, 40 %) and lower anterior resection (LAR) (15/40, 37.5 %). Among all 40 patients, the median time to first flatus passage was 2 days. The median time to soft diet resumption was 4 days. The median post operative hospital stay was 7 days. The overall complication rate was 20 % (8/40 patients), of which most of the complications were mild, although one laparotomy was required to check for post-operative bleeding. There was no 30-day hospital mortality, nor conversion to open surgery and laparoscopy.

**Conclusion:**

We present our preliminary experiences of robotic colorectal surgery and demonstrate that robotic colorectal surgery is a safe and feasible surgery even when conducted by laparoscopic surgeons with limited experience.

## Background

The main purpose and principle of surgical management for colorectal cancers (CRC) is to excise the intestinal segment bearing the tumor mass with adequate surgical margins. Therefore, the extent of surgical resection includes both the intestine segment bearing the tumor mass and locoregional lymphatic tissue. The evolution of surgical approaches has progressed gradually from the open method (i.e. laparotomy) to minimally invasive methods. Since the first laparoscopic colectomy was reported in 1991 [1], laparoscopic colorectal surgeries have gradually been performed in more and more institutions worldwide because this approach has been proven to be beneficial to patients, including better short-term outcomes and equivalent oncology safety in comparison to open surgeries [2-6]. However, laparoscopic rectal surgery with total mesorectal excision (TME) is a more challenging procedure than laparoscopic colon surgery. The narrow space of the pelvis and rigid laparoscopic instruments with limited dexterity and range of motion make TME more difficult and, as a result, require a longer learning curve for surgeons who preform laparoscopic rectal surgeries. Moreover, hand tremor of the camera-holding assistants and the resulting instability of two-dimensional (2D) visualization images are other limitations hindering conventional laparoscopic rectal surgery.

The robotic system (da Vinci® Surgical System, Intuitive Surgical, Inc., Sunnyvale, CA, USA) used in this study has three-dimensional (3D), enhanced high-definition vision with up to 10-x magnification. In addition, the Endowrist® instruments of the system are designed to provide the surgeon with natural dexterity and a range of motion far greater than that of the human hand. Moreover, the surgeon-controlled camera platform, ergonomic setting console, and stable traction provided by the robotic arm are also potential advantages of the robotic system. Since the first robotic colon surgery was reported in 2002 [7], such robotic systems have been expected to overcome the disadvantages of conventional laparoscopic colorectal surgery and improve the clinical outcomes of minimally invasive surgeries for colorectal patients. In addition, the learning curve for robotic colorectal surgery is also reported to be shorter than that for conventional laparoscopic colorectal surgery [8,9].

We started to perform robotic surgery for CRC with the da Vinci® Surgical System in May 2013, and a total of forty CRC patients had received robotic surgery by September 2014. The purpose of this study is to present our experiences and the subsequent peri-operative outcomes for the 40 patients with CRC treated by robotic colorectal operations.

## Methods

### Patients

The present study included 40 consecutive patients with CRC who received robotic surgery conducted using the da Vinci® Si at a single-institution between May 2013 and September 2014. The robotic surgeries were performed by surgeon Jaw-Yuan Wang and assistant surgeon Ching-Wen Huang. The present study was approved by the Institutional Review Board of the Kaohsiung Medical University Hospital. Informed consent was obtained from each patient before the respective robotic surgery was conducted.

All the patients routinely received a pre-operative colonoscopy image evaluation and an abdominal and pelvic computed tomography (CT) scan. Patients with T3/T4 or N+ rectal cancer received pre-operative concurrent chemoradiotherapy (CCRT) with FOLFOX regimen or oral fluoropyrimidines, capecitabine (Xeloda®, Roche Pharmaceuticals, Basel, Switzerland) or tegafur-uracil (UFUR®, combined in a 1:4 molar ratio, TTY Biopharm, Taipei, Taiwan). Patients with stage IV rectal cancer received pre-operative CCRT with FOLFIRI plus bevacizumab (Avastin®, Roche Pharmaceuticals, Basel, Switzerland) or FOLFIRI plus Cetuximab (Erbitux®, ImClone Systems Inc, New York, NY, and Bristol-Myers Squibb Co, Princeton, NJ), and long-course radiotherapy (total 50.4 Gy in 25 fractions) was administrated. There were 3 patients with previous pelvic surgery.

Available clinicopathological features and perioperative parameters included: age of diagnosis, sex, tumor location, histological type, TNM classification, vascular invasion, perineural invasion, preoperative and postoperative serum carcinoembroyonic antigen (CEA) levels, and body mass index (BMI). The TNM classification was defined according to the criteria of the American Joint Commission on Cancer/International Union Against Cancer (AJCC/UICC). Perioperative outcomes included surgical procedures, docking time, console time, operation time, estimated blood loss, time to first flatus passage, time to soft diet resumption, post operative hospital stay, and post-operative 1st day visual analogue scale (VAS) pain score.

Patients’ clinical outcomes and survival statuses were regularly followed up. The protocols of postoperative surveillance that were used followed the NCCN (National Comprehensive Cancer Network) guidelines (version 2, 2014). A patient history and physical examination were conducted every 3 months for 2 years, and then every 6 months for a total of 5 years. A CEA (carcinoembryonic antigen) test is recommended every 3 months for 2 years, then every 6 months for a total of 5 years for patients with stage III disease and those with stage II disease at a high risk for recurrence. A colonoscopy is recommended at approximately 1 year after resection. A repeat colonoscopy is typically recommended at 3 years, and then every 5 years thereafter, unless a follow-up colonoscopy indicates advanced adenoma (villous polyp, polyp >1 cm, or high-grade dysplasia), in which case colonoscopy should be repeated at 1 year. Chest CT scan, abdominal CT scan, and pelvic CT scan are recommended annually for up to 5 years in patients with stage III disease and those with stage II disease at a high risk for recurrence.

### Surgical procedure

#### Left-sided colon and rectum

**Fig. 1:**
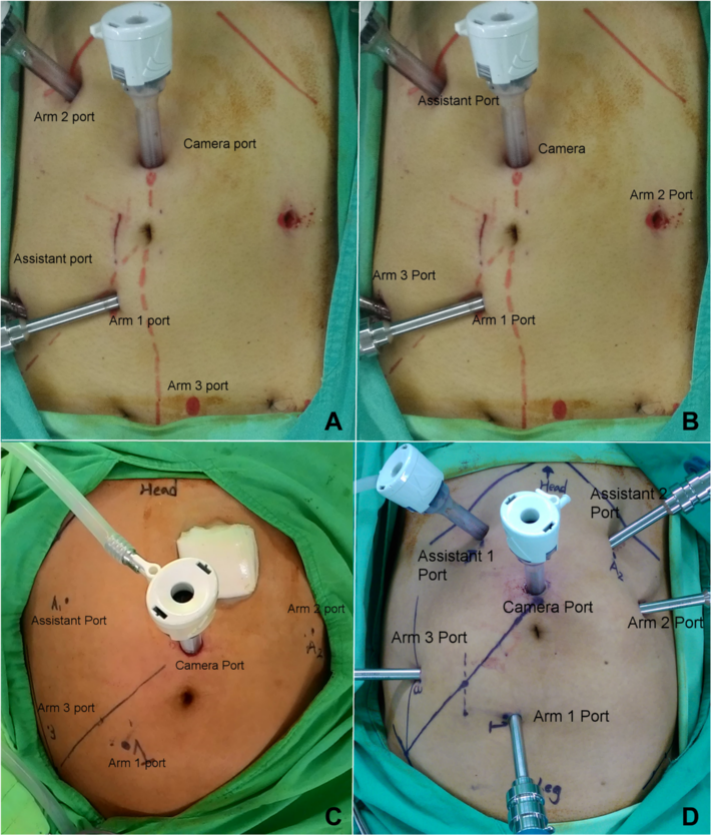
Port positions (**a**) Stage 1 procedure of dual docking. (**b**) Stage 2 procedure of dual docking. (**c**) Single docking with the five-port technique. (**d**) Single docking with the six-port technique

Initially, dual docking with the six-port technique was set-up (Fig. 1a and b). After the induction of general anesthesia, the patient was placed in a modified lithotomy position and tilted right side down at 15°. Both legs were abducted with the help of adjustable stirrups, and both arms were placed alongside the body. Pneumoperitoneum, the insufflation of the abdomen with CO_2_, was established using a Veress needle inserted through a 1-mm umbilical incision. The insufflator was set to a pressure of 12–14 mmHg. The dual docking technique with six ports was used. A 12-mm optical port for the camera was inserted via a midline skin incision 2 cm superior to the umbilicus. A line from the approximate location of the splenic flexure across the camera port down to the right anterior superior iliac spine (ASIS) was made. One 8-mm port (Arm 1 port) was placed under direct vision approximately 2 cm inferior to the line and slightly medial to the right mid-clavicular line (MCL). One 8-mm port was placed under direct vision right lateral 8 cm from the Arm 1 port. This port was used as an assistant port during the stage 1 procedure and as the Arm 3 port during the stage 2 procedure. One 8-mm port was placed under direct vision at the right MCL, approximately 4 cm inferior to the right costal margin. This port was used as the Arm 2 port during the stage 1 procedure and as an assistant port during the stage 2 procedure. One 8-mm port was placed under direct vision just above the pubic bone, 2 cm to the left of the midline, as the Arm 3 port during the stage 1 procedure. Another 8-mm port was placed under direct vision left laterally 8 cm from umbilicus, as an assistant port during the stage 1 procedure and as the Arm 2 port during the stage 2 procedure (Fig. 1a and b). In female patients, the uterus was attached to the abdominal wall by using a percutaneously inserted 2/0 Vicryl® suture with a straight needle. A monopolar permanent cautery spatula (Intuitive Surgical, Inc., Sunnyvale, CA, USA) was used in Arm 1, a Maryland bipolar forceps (Intuitive Surgical, Inc., Sunnyvale, CA, USA) was used in Arm 2, and a double fenestrated grasper (Intuitive Surgical, Inc., Sunnyvale, CA, USA) was used in Arm 3. During stage 1, the da Vinci® Si Surgical System was docked over the patient’s left flank. We used medial to lateral dissection to ligate and divide the inferior mesenteric vessels (artery and vein). First, we started to perform peritoneal incision at the level of the sacral promontory by using the monopolar permanent cautery spatula on Arm 1. Then, the dissection was extended upward and downward. Afterward, the inferior mesenteric artery (IMA) was identified and ligated near the origin by using endo clips (Hem-O-Lok, Weck Closure Systems, NC). When the IMA was too wide to be liagted by endo clip (Hem-O-LoK) alone, we first ligated the IMA by 3–0 silk and then applied the Hem-O-Lok. The inferior mesenteric vein (IMV) was also identified, but was not ligated immediately. If there was tension during the colonic anastomosis, the IMV would be ligated by using endo clips and divided (Hem-O-LoK). During the stage 2, the da Vinci® Si Surgical System was docked over the patient’s left hip. The rectum was mobilized with total mesorectal excision (TME) down to the pelvic floor by using the monopolar permanent cautery spatula.

In the initial six cases, splenic flexure of the colon was routinely taken down by the dual docking technique. Subsequently, we changed to single docking with the five-port (Fig. 1c) or six-port (Fig. 1d) technique because the take down of the splenic flexure was not routinely performed and was dependent on the tension of the anastomosis. The sites of the camera port, Arm 1 port, Arm 3 port, and assistant port were the same as the ones used in stage 2 of the dual docking technique.

**Fig. 2:**
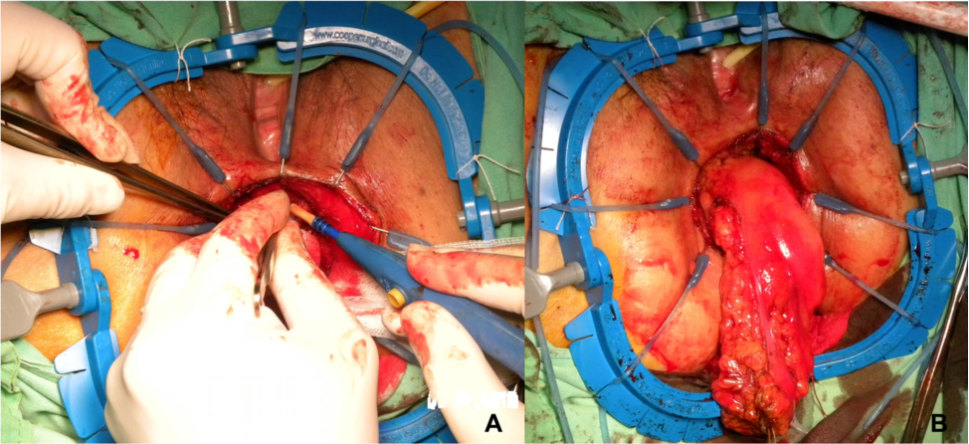
Intersphincteric resection (ISR) and specimen extraction. (**a**) ISR. (**b**) Transanal extraction and resection of specimen (natural orifice specimen extraction)

After the completion of mobilization of the sigmoid or descending colon and mesocolon and entire rectum and TME, the da Vinci® Si Surgical System was undocked. In cases of descending colon cancers or sigmoid colon cancers, the camera port wound was extended to a 2–3 cm length, and the wound protector/retractor (Alexis®, Applied Medical, CA) was used to protect the wound sites. The proximal colon was extracted through this wound and was transected by using a GIA stapler (DST Series™ GIA™ Single Use Reloadable Staplers, Covidien) at first. Then, the distal colon was transected in the same way. Hand-sewn end-to-end anastomosis was performed extracorporeally. For a tumor located in the upper and mid rectum, the surgical procedure used was low anterior resection (LAR) with the double-stapled technique. The rectum was divided by the assistant using an Endo GIA roticulator stapler (Endo GIA™ Reinforced Reload with Tri-Staple™ Technology, Covidien) with one to three 60-mm purple loads. The specimen was extracted through the extended camera port wound with the Alexis® wound proctor and divided. Pneumoperitoneum was re-established and the anastomosis was performed laparoscopically using a circular EEA stapler. In cases of low rectal cancers, the surgical procedure used was intersphincteric resection (ISR). The Lone Star Retractor System® (Lone Star Medical Products Inc., Houston, TX) was used for ISR (Fig. 2). Then, the specimen was extracted and resected transanally (natural orifice specimen extraction). Coloanal anastomosis was performed using the hand-sewn method. A loop colostomy of transverse colon was created. Finally, the traditional laparoscope was used to check any bleeding in the abdominal cavity. A drain tube was placed into the pelvic cavity.

#### Right-sided colon

**Fig. 3:**
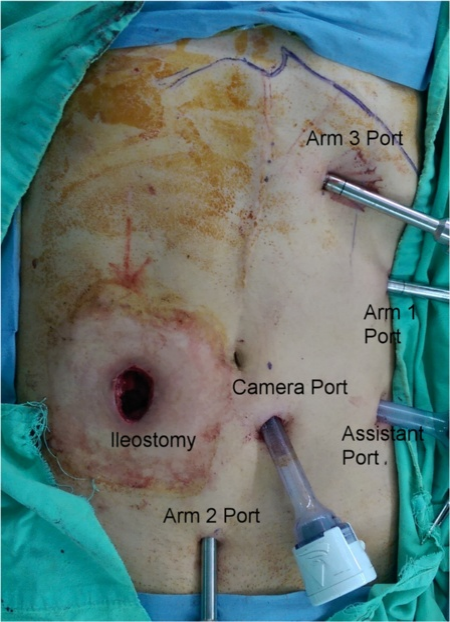
Port positions during robotic right hemicolectomy

After the induction of general anesthesia, the patient was placed in the Trendelenburg position at 10° - 15° and tilted left side down at 15°. Both legs were abducted with the help of adjustable stirrups, and both arms were placed alongside the body. Pneumoperitoneum was performed described in the above procedure. The insufflator was set to a pressure of 12–14 mmHg. A 12-mm optical port for the camera was inserted via a skin incision located 2 cm inferior to the umbilicus and 2 cm left lateral to the midline. One 8-mm port was placed under direct vision in the left upper quadrant, 2 cm lateral to the MCL and 4 cm inferior to the costal margin, and served as the Arm 1 port. One 8-mm port was placed under direct vision on the midline, 4 cm from the symphysis pubis, and served as the Arm 2 port. One 8-mm port was placed under direct vision just 4–6 cm inferior to the xiphoid process and 2 cm off midline into the left-upper quadrant, and this port served as the Arm 3 port. One 12-mm port was placed under direct vision in the left-lower quadrant, slightly inferior to the left spinoumbilical line (SUL) and slightly lateral to the left MCL, and served as an assistant port (Fig. 3). The da Vinci® Si Surgical System was docked over the patient’s right shoulder. We used inferior to superior dissection to ligate and divide the ileocolic vessels (artery and vein), right colic vessels (artery and vein), and right branch of the middle colic vessels (artery and vein, as necessary). After completion of mobilization of the ileum, cecum, ascending colon, and proximal transverse colon, the da Vinci® Si Surgical System was undocked. The camera port wound was extended to a 2–3 cm length, and the Alexis® wound proctor was used to protect the wound sites. The specimen was extracted through this wound and was transected. Then, hand-sewn end-to-end anastomosis was performed extracorporeally.

### Statistical analysis

All data were statistically analyzed using the Statistical Package for the Social Sciences, version 19.0 (SPSS Inc., Chicago, IL). The docking time was defined as the time required to position the robot and secure the robotic arms to the corresponding port sites. The console time was defined as the total time during which the surgeon performed any procedure using the robotic system. The operation time was defined as the time between the initial skin incision and the completion of wound closure. The 7-case simple moving average method was used to analyze the learning curve as indicated by the various console times and operation times.

## Results

### Characteristics of 40 Patients with CRC

The clinical and pathological data regarding the 40 patients with CRC are summarized in Table 1. Of the 40 patients with CRC, 39 patients had adenocarcinoma and one patient had rectal neuroendocrine tumor. Of the 40 patients with CRC, 33 patients (82.5 %) had rectal cancer, 4 patients (10.0 %) had sigmoid colon cancer, 2 patients (5.0 %) had descending colon cancer, and 1 patient (2.5 %) had ascending colon cancer. Of the 33 patients with rectal cancers, 22 (66.7 %) patients had undergone pre-operative CCRT, and pathological complete response (pCR) was noted in 5 patients (22.7 %). The patient with the rectal neuroendocrine tumor (pT2N1M0, stage IIIA, 5 cm from the anal verge, 2 cm in size) underwent LAR with the double-stapled technique. The mean age of the 40 patients was 60.00 ± 13.22 (range, 32–89) years old. There were 21 male and 19 female patients. The majority of tumors were <5 cm (90.0 %) and the mean tumor size was 2.58 cm. The median number of retrieved lymph nodes was 9 (range, 0–22) in all patients, 7 in patients with pre-operative CCRT (range, 0–16), and 12 in patients without pre-operative CCRT (range, 7–22). The mean body mass index (BMI) was 23.37 kg/m^2^ (range, 17.20 - 34.02). There was no conversion to open surgery and no conversion to laparoscopy.

**Table 1:** Baseline characteristics of 40 patients who underwent robotic colorectal surgery

Characteristic	
Age (years, mean ± SD) (range)	60.00 ± 13.22 (32 – 89)
Gender	
Female	21 (52.5 %)
Male	19 (47.5 %)
Tumor size	
<5 cm	36 (90.0 %)
≥5 cm	4 (10.0 %)
Tumor size (cm, mean ± SD) (range)	2.58 ± 1.81 (0 – 8)
Tumor location	
Ascending colon	1 (2.5 %)
Descending colon	2 (5.0 %)
Sigmoid colon	4 (10.0 %)
Rectum	33 (82.5 %)
Histology	
Tis	2 (5.0 %)
Well	5 (12.5 %)
Moderately	33 (82.5 %)
AJCC Stage^a^	
0	7 (17.5 %)
I	7 (17.5 %)
II	7 (17.5 %)
III	14 (35.0 %)
IV	5 (12.5 %)
Tumor depth	
T0	6 (15.0 %)
Tis	2 (5.0 %)
T1	3 (7.5 %)
T2	13 (32.5 %)
T3	16 (40.0 %)
Lymph Node metastasis	
N0	23 (57.5 %)
N1	15 (37.5 %)
N2	2 (5 %)
Retrieved Lymph Node (median) (range)	
All patients	9 (0–22)
Patients with pre-op CCRT^b^	7 (0–16)
Patients with without pre-op CCRT^b^	12 (5–22)
Vascular invasion	
No	31 (77.5 %)
Yes	9 (22.5 %)
Perineural invasion	
No	34 (85.0 %)
Yes	6 (15.0 %)
Pre-op serum CEA^c^ level	
<5 ng/ml	20 (50.5 %)
≥5 ng/ml	20 (50.0 %)
Post-op serum CEA^c^ level	
<5 ng/ml	29 (75.0 %)
≥5 ng/ml	11 (25.0 %)
ASA^d^ classification	
II	24 (60.0 %)
III	16 (40.0 %)
Diabetes mellitus	
Yes	10 (25.0 %)
No	30 (75.0 %)
BMI^e^ kg/m^2^ (range)	23.77 ± 3.86 (17.20 – 34.02)

^a^AJCC American Joint Commission on Cancer; ^b^Concurrent chemoradiotherapy

^c^CEA Carcinoembryonic antigen; ^d^ASA American Society of Anesthesiologists

^e^BMI Body mass index

### Peri-operative outcomes of 40 patients with CRC

The peri-operative outcomes for the 40 patients are summarized in Table 2. The two most frequent surgical procedures were ISR with coloanal anastomosis (16/40, 40 %) and LAR (15/40, 37.5 %). Protective loop transverse colostomy was performed in 18 patients, including 16 patients who underwent ISR and 2 patients who underwent LAR. For one patient, anastomosis leakage was noted 6 days after robotic LAR and loop transverse colostomy was performed. In another patient with rectal cancer, anastomotic leakage was noted during the operation via an intraoperative dye test [10], and thereafter loop transverse colostomy was performed. The mean docking time was 7.38 min. The mean console time was 264.13 min. For all 40 patients, the mean operation time was 492.00 min. The median estimated blood loss (including tissue fluid after CCRT) was 150 ml. The mean time to first flatus passage was 2 days. The median time to soft diet resumption was 4 days. The media post-operative hospital stay was 7 days (range, 5–32). The median post-operative 1st day pain score (VAS score) was 3.

**Table 2:** Peri-operative outcomes of 40 patients who underwent robotic colorectal surgery

Parameters	
Procedure	
Right hemicolectomy	1 (2.5 %)
Left hemicolectomy	1 (2.5 %)
AR^a^	5 (12.5 %)
LAR^b^	15 (37.5 %)
ISR^c^ with coloanal anastomosis	16 (40.0 %)
APR^d^	2 (5.0 %)
Docking Time (min, mean ± SD) (range)	7.38 ± 4.05 (3 – 22)
Console Time (min, mean ± SD) (range)	264.13 ± 76.57 (109 – 527)
Operation Time (min, mean ± SD) (range)	492.00 ± 118.69 (270 – 825)
Estimated blood loss (mL, Median)^e^	150 (20 – 500)
Time to first flatus passage (day) (Median, range)	2 (1–9)
Time to soft diet resumption (day) (Median, range)	4 (3–13)
Post-operative hospital stay (day) (Median, range)	7 (5–32)
Post-operative 1st day pain score (VAS^f^ score) (Median, range)	3 (1–8)

^a^Anterior resection

^b^Low anterior resection

^c^intersphincteric resection

^d^Abdominoperineal resection

^e^Including tissue fluid

^f^VAS visual analogue scale

### Post-operative complications

The post-operative complications are summarized in Table 3. Post-operative complications were noted in 14 episodes, of which occurred in 8 of 40 patients. One patient developed intra-abdominal bleeding after robotic surgery. A laparotomy was then performed because of unstable hemodynamic condition, and bleeding from the mesocolon was noted. Four patients developed intra-abdominal abscess and CT-guided pig-tail drainage was performed in 2 of those patients. Anastomosis leakage was noted in a patient with rectal cancer undergoing LAR with double-stapled technique and loop transverse colostomy was performed. Two patients developed stenosis of coloanal anastomosis and underwent dilation with colonoscope. Post-operative ileus, urinary tract complications, and pulmonary complications were all grade I according to the Clavien-Dindo classification [11] and were improved after conservative treatment. Moreover, there was no 30-day hospital mortality.

**Table 3:** Post-operative complications in 40 patients who underwent robotic colorectal surgery

Complication	Number	Management
Post-operative bleeding	1	Laparotomy
Intra-abdominal abscess	4	2: conservative treatment 2: CT-guided pig-tail drainage
Anastomotic leakage	1	Loop transverse colostomy
Coloanal anastomosis stenosis	2	Dilation with colonoscope
Ileus	1	Conservative treatment
Urinary tract complication	3	Conservative treatment
Urine retention	1	
Infection	2	
Pulmonary complication	2	Conservative treatment
Total	14	

### Learning curve of robotic CRC surgery

**Fig. 4:**
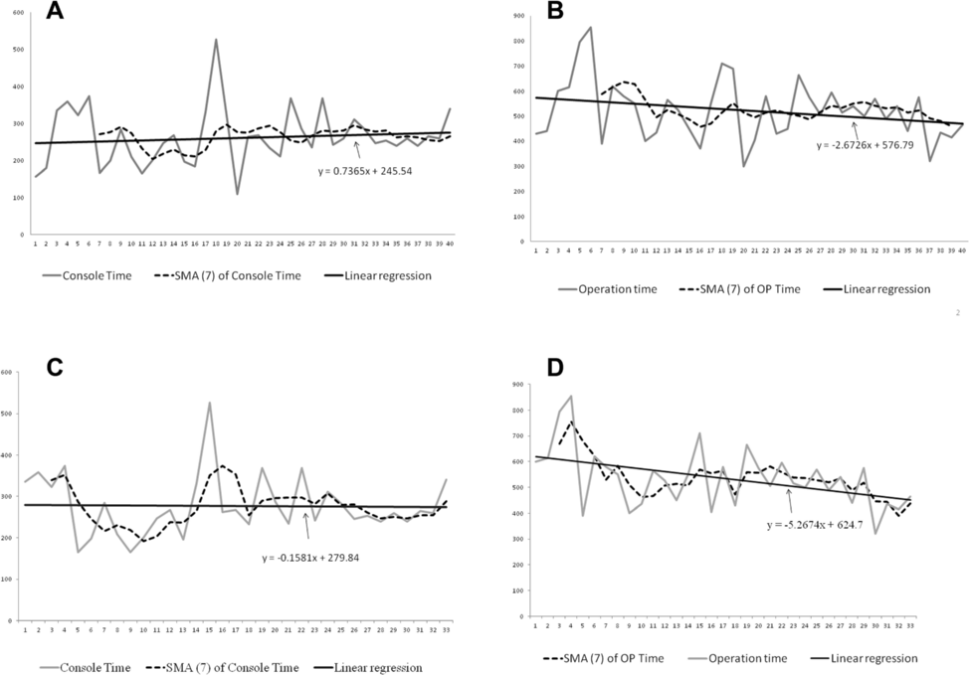
Learning curves for robotic colorectal surgery. **a** Console time; **b** Operation time. Learning curves for robotic rectal surgery. **c** Console time; **d** Operation time

The learning curves in terms of console times and operative times are shown in Fig. 4. Severe adhesions were noted in the cases numbered 18, 25, and 28 in all CRC (Fig. 4a), or numbered 15, 19, 22 in rectal cancers (Fig. 4c) because they had previous pelvic surgery. Enterolysis was performed simultaneously. Therefore, the corresponding operation times were longer. The 7-case simple moving average method was used to analyze the learning curve of console time and operation time. The first plateau of console time was noted after 23 patients. The linear regression revealed a trend of increasing console time. The first plateau of operation time was noted after 31 patients. However, the linear regression revealed a trend of decreasing operation time. As for robotic rectal surgery, the first plateau of console time was noted after 15 patients. The first plateau of operation time was noted after 21 patients. Moreover, the linear regression revealed a decreasing trend for both console time and operation time.

## Discussion

In May 2013, we performed the first robotic surgery in a patient with descending colon cancer. The console time was 156 min and the operation time was 400 min. The second case was a patient with sigmoid colon cancer. The console time was 180 min and the operation time was 400 min. The third case was a 38 year-old female patient with rectal cancer, 3 cm above the anal verge. She received local resection and radiation therapy in October 2012 at another hospital. Local recurrent tumor was noted and she received robotic surgery (ISR with coloanal anastomosis) at our hospital in May 2014. The console time was 335 min and the operation time was 570 min. The pathological report was ypT0N1M0 (UICC stage IIIA).

Previous studies have reported potentially significant benefits in rectal surgery and suggested rectal cancer as a good indication for robotic surgery [12-15]. Therefore, following this indication, the majority of the patients included in this study were rectal cancer patients (33/40, 82.5 %). Moreover, of the 33 patients with rectal cancers, 22 patients with locally advanced stage underwent pre-operative CCRT. Therefore, ISR with coloanal anastomosis and LAR were the two most frequently performed surgical procedures in our hospital.

Before we began performing robotic surgeries in May 2013, each surgeon involved in this study (Wang JY and assistant surgeon Huang CW) had performed fewer than 40 laparoscopic colorectal surgeries, and we had only performed 40 laparoscopic colorectal surgeries in total. In the present study, our mean docking time was 7.38 min, which was comparable to time reported by Sng *et al*. [16]. Our mean console time was 264.13 min, which was longer than the console times reported in previous studies [16-18]. However, a trend of decreasing console time was noted for rectal surgery. In previous studies, the reported operation times for robotic colon surgery and rectal surgery were 197 to 383 min and 178 to 519 min, respectively [19,20]. Our mean operation time was 492.00 min. The reasons for this longer mean console time and operation time would be as follows. First, of the 40 patients in the present study, 82.5 % of thepatients had rectal cancer and received ISR with coloanal anastomosis, LAR, or APR (abdominoperineal resection). The second reason for our longer operation time was that there were 3 cases with previous pelvic surgery and robotic enterolysis was performed simultaneously. Therefore, our mean console time and operation time were longer than those reported in some previous studies, but similar to those reported by Kuo *et al*. [20]. Moreover, a trend of decreasing operation time was noted for both colorectal surgeries and rectal surgeries.

The median estimated blood loss of 150 ml (Range: 20 – 500) in the present study was more than those for robotic colon surgery reported in the literature, but comparable to those for robotic rectal surgery reported in the literature [19]. Because 55 % of our patients received pre-operative CCRT, the tissues of these patients were very edematous during their operations. Much fluid was noted during tissue dissection and summed into the estimated blood loss. We were not able to deduct the amount of tissue fluid from the amount of estimated blood loss. Therefore, the actual blood loss in the present study should be far less than the estimated loss. Moreover, no patient received a blood transfusion during the operation.

The total complication rate was 14 episodes (8 of 40 patients) in the present study, which was comparable to the rates for robotic rectal surgery reported in previous studies [19]. Only one patient underwent a laparotomy to check for intra-abdominal bleeding. Anastomotic leakage was only noted in one patient, and diversion colostomy was created accordingly. CT-guided pig-tail drainage was performed in 2 patients with intra-abdominal abscess. Other complications were mild and conservative treatment was administrated. No 30-day hospital mortality was noted in the present study.

The docking method used in robotic colorectal surgery varies from surgeon to surgeon. We used the dual docking method initially and then changed to the single docking method. We did not take down the splenic flexure routinely. When it was necessary to take down the splenic flexure, we reset the setting of the console to allow the surgeon to control the different robotic arms, rather than re-docking the surgical cart. We had ever encountered arm collision when we performed pelvic dissection, and we subsequently changed to use monopolar permanent cautery spatula in the Arm 3 and double fenestrated grasper in the Arm 1 to reduce the arm collision.

The robotic colorectal surgeries were performed only by two surgeons (Wang JY and assistant surgeon Huang CW). However, this could also be viewed as an advantage because the methods used in specimen extraction, anastomosis, and the post-operative management of patients were more consistent than they would have been if more surgeons has been involved. Therefore, the results of the statistical analysis were more uniform.

However, there are some limitations to the present study. First, the present study is a single-institution retrospective study. Secondly, we did not analyze the individual results of colon cancers and rectal cancers because the sample size was relatively small in the present study.

## Conclusion

We present our preliminary experiences with robotic colorectal surgery in regard to port placement, docking method, and the use of robotic instruments. In the present study, we report a longer mean console time but, comparable mean docking time, mean operation time, post-operative recovery, and complication rate to the previous reports in the literature. Moreover, we also demonstrate that improvements of console time and operation time can be achieved with increased case numbers. In summary, robotic colorectal surgery is a safe and feasible surgery even when conducted by a laparoscopic surgeon with limited experience.

## Abbreviations

CRC: Colorectal cancer
CCRT: Concurrent chemoradiotherap
ISR: Intersphicteric resection
LAR: Lower anterior resection
TME: Total mesorectal excision
VAS: Visual analogue scale
MCL: Mid-clavicular line
IMA: Inferior mesentaric artery
IMV: Inferior mesentaric vein
pCR: Pathological complete response
BMI: Body mass index
APR: Abdominoperineal resection
